# Adsorption Characterization of Cu(II) and Cd(II) by a Magnetite–Chitosan Composite: Kinetic, Thermodynamic and Equilibrium Studies

**DOI:** 10.3390/polym15122710

**Published:** 2023-06-16

**Authors:** Chao Hu, Zuhong Zheng, Mengyao Huang, Fan Yang, Xuewei Wu, Aiqun Zhang

**Affiliations:** 1 Hubei Province Research Center of Engineering Technology for Utilization of Botanical Functional Ingredients, Hubei Engineering University, Xiaogan 432000, China; 2 College of Life Science and Technology, Hubei Engineering University, Xiaogan 432000, China

**Keywords:** chitosan, magnetite, adsorption, copper, cadmium

## Abstract

Optimizing the use of magnetite–chitosan composites for heavy metal adsorption has been of great interest due to their environmental friendliness. To gain insights into their potential with green synthesis, this study analyzed one of these composites through X-ray diffraction, Fourier-transform infrared spectroscopy and scanning electron microscopy. Adsorption properties were then explored via static experiments to evaluate the pH dependence, isotherms, kinetics, thermodynamics and regeneration adsorption of Cu(II) and Cd(II). Results disclosed that the optimum pH of adsorption was 5.0, the equilibrium time was about 10 min, and the capacity for Cu(II) and Cd(II) reached 26.28 and 18.67 mg/g, respectively. The adsorption amount of cations increased with temperature from 25 °C to 35 °C and decreased with further increase in temperature from 40 °C to 50 °C, which might be related to the unfolding of chitosan; the adsorption capacity was above 80% of the initial value after two regenerations and about 60% after five regenerations. The composite has a relatively rough outer surface, but its inner surface and porosity are not obvious; it has functional groups of magnetite and chitosan, and chitosan might dominate the adsorption. Consequently, this research proposes the value of maintaining green synthesis research to further optimize the composite system of heavy metal adsorption.

## 1. Introduction

Heavy metal cations released into the environment with industrial and farming pollution sources, such as chemicals and fertilizers, often result in an increase in heavy metal concentrations in water resources and may ultimately accumulate through the food chain and pose a grave danger to both human health and ecological security [1]. Since heavy metals cannot be biodegraded and will not be eliminated through chemical processes, they may move, transform and/or accumulate in water bodies or soil [2]. Due to their distinguishing features and speciation transformation, for metals such as Hg and Pb, and concentration passivation, for metals such as Cd and Cu, contamination caused by heavy metal cations has become an area of increasing concern in the research field [3,4,5].

Due to its features such as cost-effectiveness, innovation and the ability to remove heavy metal cations, an adsorption method utilizing nanomaterials has become widely used for treating heavy metal contamination in soil and water environments [3,6,7]. Recently, the focus has shifted to cost-efficient cutting-edge nanomaterials for water and wastewater purification, such as zeolites, carbonaceous substances, polymer-based materials, chitosan, ferrite, magnetite, metal oxides and so on [8,9]. Out of all of these, biomass has been extensively explored due to its green, natural, stable and competent properties [10,11,12]. In particular, research on chitosan-based natural adsorbents has been on the rise [4,13,14].

Chitosan is the deacetylation product of chitin, and chitin is a natural substance second only to cellulose in nature reserves. Therefore, chitosan is a natural amino polysaccharide with excellent bio-affinity and non-toxicity and is easy to obtain and modify [15,16]. It can interact with Hg^2+^, Ni^2+^, Pb^2+^, Cd^2+^, Zn^2+^, Cu^2+^ and other transition element ions to form stable chelates [17]. Therefore, in the research field of heavy metal adsorption, chitosan has attracted much attention.

Despite its precious features, chitosan’s application has been restricted in lots of aqueous milieus, partly attributed to its meager mechanical strength and acid vulnerability. To ensure the versatility of its valuable attributes and improve its usage range and suitability for various conditions, abundant studies have been conducted, focusing on the alteration and conjugation of chitosan. With regard to water and wastewater treatment, natural minerals and organic matters are habitually used to modify or compound chitosan, thereby raising its compression severity and sediment velocity, with the goal of experiencing commendable adsorption and mud-water separation [18].

Combining hydroxyapatite with chitosan significantly enhanced the mechanical strength of the composite, up to 2.40 ± 0.51 MPa. Dissolution tests confirmed the cohesion and mechanical stability of the composite [19]. Comparing the combination of activated carbon with chitosan, bentonite (montmorillonite) and chitosan-amended soil had the highest significance in reducing Ni distribution in roots, shoots and grains and DTPA (diethylenetriaminepentaacetic acid) extractable fractions of Ni in lentils [20]. The Fe(III)–chitosan microbeads showed adsorption capacities of 34.15 and 32.27 mg/g for Cr(VI) and PO_4_^3−^ ions at a pH of 3 and a more than 80% detoxification rate. The adsorption process of Cr(VI) and PO_4_^3−^ ions is spontaneous and exothermic, with pseudo-second-order kinetics for both adsorbates [21]. Selective flotation of pyrite in galena could be achieved by 400 g/t chitosan (10 kDa), 1600 g/t ethyl xanthate and 100 g/t terpineol. Zeta potential and contact angle measurement showed strong adsorption of chitosan (10 kDa) on galena but very weak adsorption on pyrite at a dose of 400 g/t, which indicated that the interaction between chitosan and metal is worthy of further study [22].

Out of the materials proposed for combination with chitosan, magnetite has also received much attention from researchers due to its separation aptitude in aqueous environments, induced by an outside magnetite exertion field [4,23]. Commonly adopted microemulsion and hydrothermal approaches can produce components with a particle size of 20–45 nm or 10–50 nm, herein referred to as the magnetite–chitosan nanocomposite [24,25], which has been regarded as a superb recyclable adsorbent for heavy metals [25].

However, most research concerning the magnetite–chitosan composite has mainly been concentrated on morphology shifts, such as the bead [15], microsphere [16,25] or film [26], or on agent add-ons, such as kaolinite [15] and glutaraldehyde [27], as ways of enhancing their efficacy. Such research results are usually quite inconsistent, and there has not been enough in-depth study about the performance of a pure magnetite–chitosan system for cation adsorption. To begin with, incorporating many chemical species renders the adsorbent inappropriate for some “green” events. Additionally, the interrelationship between magnetite and chitosan, as well as their adhesion performance for heavy metals, should receive more attention. Since green natural materials are not endowed with highly aggressive traits, deliberating this combination’s association is important to optimize its utilization and applications [4,15,26].

In this paper, no grafting or modified reagents were used to synthesize the magnetite–chitosan composite. X-ray diffraction (XRD) and Fourier-transform infrared spectroscopy (FTIR) were utilized to characterize the chitosan–magnetite composite.

## 2. Materials and Methods

### 2.1. Materials

Low-molecular-weight chitosan (80% degree of deacetylation, 20–300 cP viscosity, CAS No. 9012-76-4) was purchased from Sigma-Aldrich (Shanghai, China). Ferrous sulfate (FeSO_4_·7H_2_O), ferric chloride (FeCl_3_·6H_2_O), sodium hydroxide (NaOH), copper nitrate (Cu(NO_3_)_2_), cadmium nitrate(Cd(NO_3_)_2_) and other chemicals were purchased from Sinopharm (Shanghai, China). Unless otherwise stated, all materials used in this study were of analytical grade. The experiment was conducted using ultrapure water (resistivity = 18.25 MΩ) at room temperature (20–25 °C).

### 2.2. Adsorbent Preparation

The magnetite–chitosan composite was synthesized by a co-precipitation method [27]. First, 5.7703 g ferric chloride and 2.7805 g ferrous sulfate (in a molar ratio of 2:1) were mixed in 200 mL ultrapure water. Then, 10 g chitosan was added to this solution on a heated mechanical stirrer (at 70 °C and 250 rpm). After standing for 3 h, 5 mol/L NaOH solution was added drop by drop with stirring (bringing the pH to 11). The black sediment obtained after centrifugation was washed with ultrapure water (until the pH of the cleaning solution was 7.0) to remove unbonded chitosan and soluble salts. The sediment was subsequently freeze-dried, passing through a 100-mesh sieve [4,15], and stored for future use.

### 2.3. Characterization of Adsorbent

The magnetite, chitosan and magnetite–chitosan composite powder patterns were recorded by X-ray Diffraction (XRD) on a Bruker D8 Cu-Ka (Billerica, MA, USA) using a step scan mode with 2θ ranges from 5 to 40°, 0.05° step increments and 5s dwell time. Energy dispersive detection (SolX, Bruker, Billerica, MA, USA) was used, without a single-light detector. 

Next, 5 mL of composite or chitosan dispersion (20 g/L) was accumulated, settled on a polished 25 mm × 2 mm ZnS transmission disc (ClearTran, International Crystal Labs, Garfield, NJ, USA) and air-dried before being heated under a lamp. Fourier-transform infrared spectroscopy (FTIR) was performed with a Spectrum 100 (PerkinElmer, Waltham, MA, USA) in transmission mode with a resolution of 2 cm^−1^. Each sample was scanned 64 times (within the range from 4000 to 700 cm^−1^) and averaged.

The scanning electron microscope (SEM, Hitachi, S4800, Chiyoda, Yokohama, Japan) and energy-dispersive X-ray spectroscopy (EDS, Horiba, EMAX x-act, Kyoto, Japan) were used to analyze the morphology and element abundance of composite powder sample. Images of the composite at magnifications of 30,000, 50,000, 100,000 and 200,000 had been taken, and the element abundance in selected areas by both surface scanning and point scanning had been recorded.

### 2.4. Adsorption Experiments

#### 2.4.1. The Effect of pH on the Adsorption

The pH of Cu(NO_3_)_2_ and Cd(NO_3_)_2_ solution (each with a concentration of 200 mg/L Cu or Cd) were adjusted to 3.0, 3.5, 4.0, 4.5, 5.0, 5.5 and 6.0, respectively. Then, 100 mL of the solutions and 0.1 g of a composite were added to a 250 mL Erlenmeyer flask solution, which was then sealed with plastic wrap and a rubber band. The flasks were shaken at 200 rpm for 1 h before 15 mL samples of the reaction solution were taken out and centrifuged at 9000 rpm for 30 min, with each treatment being repeated 3 times. Next, 10 mL of the centrifuged supernatant was taken out to measure the concentration of Cu(II) and Cd(II) on the flame atomic absorption spectrophotometer (TAS-990, Beijing General Analysis, Beijing, China). The difference between the initial and equilibrium concentrations was then used to calculate the adsorption amount in the equilibrium state.

#### 2.4.2. Adsorption Isotherms of Cu(II) and Cd(II) on Composite

Next, 100 mL of Cu(NO_3_)_2_ or Cd(NO_3_)_2_ solution at pH 5.0 (ranging from 0, 1, 2, 5, 10, 20, 50, 100, 200 mg/L Cu or Cd) was mixed with 0.1 g composite in a 250 mL Erlenmeyer flask. After shaking at a rate of 200 rpm for 1 h (repeated 3 times), 15 mL of the reaction solution was centrifuged for the measurement. The measurement and calculation were similar to the method described in Section 2.4.1. The adsorption amount and the equilibrium concentration were then used to formulate an isotherm adsorption curve. Langmuir and Freundlich’s equations were employed to fit the adsorption data [5].

The linear form of the Langmuir equation is as follows:

(1) $$\frac{1}{q_{e}}=\frac{1}{q_{m}}+\frac{1}{bq_{m}C_{e}}$$

where *q_m_* is the maximum adsorption capacity for monolayer adsorption, in mg/g; *b* is the affinity between the adsorbate and the adsorption site on the adsorbent, which is used to calculate the dimensionless separation factor *R_L_*, and the formula is as follows:

(2) $$R_{L}=\frac{1}{1+bC_{0}}$$

where *C*_0_ is the initial concentration, in mg/L; the *R_L_* value can be determined to indicate whether the adsorption reaction is reversible (>1), linear (=1), favorable (0~1) or irreversible (=0).

The Freundlich equation assumes that the adsorption process is heterogeneous, and the linear form of the equation is as follows:

(3) $$lgq_{e}=lgK_{F}+\frac{1}{n}lgC_{e}$$

where *K_F_* is the relative adsorption capacity, in mg/g; 1/*n* represents the adsorption strength of heavy metal cations on the composite, related to the affinity between the adsorbent and adsorbate.

The Dubinin–Radushkevich (D–R) equation is used to account for the effect of the porous structure of the adsorbents, and the linear form of the equation is as follows [5,28]:

(4) $$lnq_{e}=lnq_{m}-\beta \varepsilon^{2}$$

where *q_m_* is the maximum adsorption capacity, in mg/g; *β* is the adsorption energy constant, in mol^2^/kJ^2^; *ε* is the potential, which can be expressed as

(5) ε=RT ln[1+1/Ce]

where *R* is the gas constant, in kJ/mol K; *T* is the adsorption temperature, in K; *Ce* is the equilibrium concentration, in mg/L. After calculating of the *ε*, *β* can be calculated according to formula (4), and then, the adsorption free energy (kJ/mol) can be calculated using the following formula:

(6) $$E=\frac{1}{\sqrt{2\beta }}$$

#### 2.4.3. Kinetic Adsorption of Cu(II) and Cd(II) on Composite

First, 100 mL of solutions containing either Cu(NO_3_)_2_ or Cd(NO_3_)_2_ at a pH of 5.0, with a concentration of 50 mg/L of the respective cation and 0.1 g of the composite, was added to a 250 mL Erlenmeyer flask, which was then sealed with plastic wrap and rubber band. The flask was then shaken at 200 rpm before being taken out after 5–90 min. The concentrations of Cu(II) and Cd(II) in the supernatant were measured, and the amount of adsorption was calculated in accordance with Section 2.4.1. To determine the rate-controlling stage among physisorption, chemisorption and intraparticle diffusion, the adsorption results were fitted with pseudo-first-order, pseudo-second-order and intra-particle diffusion (pseudo-third-order) models, with the most effective fit corresponding to the rate-controlling stage [29,30].

The linear form of the pseudo-first-order equation is

(7) $$\log\left(q_{e}-q_{t}\right)=\log q_{e}-\frac{k_{1}t}{2.303}$$

where *k*_1_ is a constant of the pseudo-first-order equation, min^−1^; *q_t_* is the adsorption capacity at time t, in mg/g; *q_e_* is the adsorption capacity at equilibrium state, in mg/g.

The pseudo-second-order kinetic equation is

(8) $$\frac{t}{q_{t}}=\frac{1}{k_{2}qe^{2}}+\frac{t}{q_{e}}$$

where *k*_2_ is a pseudo-second-order kinetic constant, in mg/g·min.

The equation of the intraparticle diffusion model is

(9) $$q_{t}=k_{i}t^{0.5}+C_{i}\;$$

where *k_i_* is the intraparticle diffusion constant, in mg/g·min^1.5^; $C_{i}$ is the relative thickness of the boundary.

#### 2.4.4. Thermodynamic Adsorption of Cu(II) and Cd(II) on Composite

To determine the thermodynamic parameters of the adsorption of Cu(II) or Cd(II), 100 mL of Cu(NO_3_)_2_ or Cd(NO_3_)_2_ solution(containing 50 mg/L of Cu or Cd) was added to a 250 mL Erlenmeyer flask with 0.1 g of the above composite. The mixture was then shaken for 1 h at temperatures of 25, 30, 35, 40, 45, and 50 °C, respectively. After this, the concentration of Cu(II) or Cd(II) in the supernatant was measured in order to calculate the adsorption amount. The thermodynamic parameters Δ*G*^0^, Δ*H*^0^ and Δ*S*^0^ were then calculated with the following formulas [31]:

(10) $$\;K_{c}=\frac{C_{ads}}{C_{e}}\;$$

(11) $$\;△G^{0}=-RTlnK_{c}$$

(12) $$lnK_{c}=\frac{△S^{0}}{R}-\frac{△H^{0}}{RT}\;$$

where *K_c_* is the equilibrium constant; $C_{ads}$ is the absolute value of the concentration difference of the adsorbate in the equilibrium state, in mg/L; $C_{e}$ is the concentration of heavy metals left in the solution in the equilibrium state, in mg/L; *R* is the gas constant, in KJ/(mol·K); *T* is the solution temperature, in K.

#### 2.4.5. Reusability Study

After the first adsorption of 100 mL containing 200 mg/L of either Cu(NO_3_)_2_ or Cd(NO_3_)_2_ onto 0.1 mg composite, at pH 5.0, the compound was centrifuged. Then, the supernatant was discarded after the measurement, and the sediment was washed with 0.1 mol/L NaCl solution to regenerate it. This process was repeated until no Cu(II) or Cd(II) were detected. Subsequently, the sediment was further washed with water until no Cl was detected. The entire procedure of adsorption, measurement and washing was repeated seven times in order to conduct regeneration and eight times for adsorption. The percentage of adsorption amount after each regeneration, compared to the first adsorption amount, was used for further data analysis.

### 2.5. Data Processing

Microsoft Excel 2013 was utilized for recording and computing data; Origin 8.0 (OriginLab CorporationOne, Northampton, MA, USA) was employed for executing the fitting and plotting; and IBM SPSS Statistics 25 was utilized for conducting significance analysis.

## 3. Results and Discussion

### 3.1. Morphology and Surface Functional Groups of the Composite

**Figure 1:**
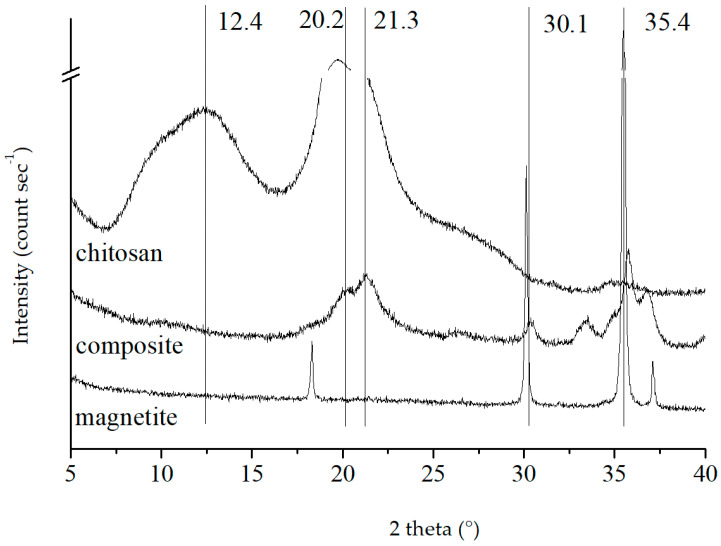
XRD patterns of magnetite, chitosan and magnetite–chitosan composite.

In the XRD pattern of magnetite in Figure 1, the characteristic peak positions of magnetite at 2θ values of 30.1 and 35.4 (d_220_, d_311_) were observed [27], corresponding to the interplanar distances of 29.66 and 25.34 nm, suggesting the formation of magnetite in the composite. The Debye–Scherrer equation indicated that the magnetite particles comprised a cubic inverse spinel structure with an average size of 48 nm [32]. The characteristic peak of chitosan at 2θ = 10.2° (d_020_) was obscured by a broad peak at 12.4°. However, the peak of chitosan at 20.2° was still notable. This broad peak encompassed two characteristic peaks at about 19° and 21.3° (d_110_ and d_120_) around 20° [33]. In the composite pattern, the 12.4° peak of chitosan was hidden, whereas the peaks at 21.3° remained, disclosing that chitosan underwent molecular chain deformation during the binding of magnetite but without any structural alteration (d_120_ unchanged). Even though the intensity of the magnetite characteristic peak decreased, there was no evidence of any red shift or blue shift, suggesting that magnetite and chitosan had successfully recombined. On the other hand, the mineral structure of magnetite remained unchanged.

**Figure 2:**
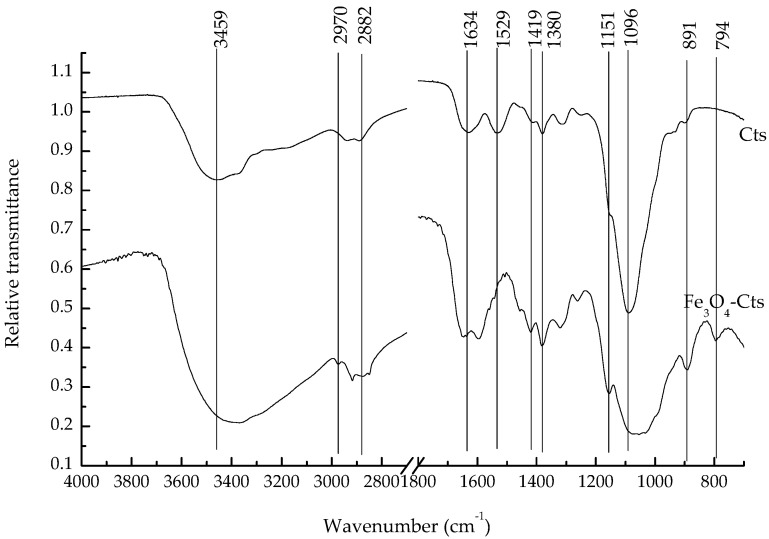
FTIR spectra of chitosan and magnetite–chitosan composite.

As is illustrated in Figure 2, the broad peak at 3459 cm^−1^ was attributed to the stretching vibration of O-H and N-H in chitosan, which shifted to 2500–3250 cm^−1^ in the composite (with the lowest point at 3370 cm^−1^), which corresponded to the stretching vibration of NH. The symmetric and asymmetric stretching vibration of amide II on chitosan appeared at 2970–2940 cm^−1^, with the methylene stretching vibration observed at 2882 cm^−1^. The bending vibration of amide Ⅰ was observed at 1634 cm^−1^ and that of amide II at 1529 cm^−1^. The symmetrical stretching vibration of -COO at 1419 cm^−1^ and the CH bending vibration at 1380 cm^−1^ were also observed, while the bending vibration of the 1–4-glycosidic bond on chitosan was shown as the unique broad peak at 1151 cm^−1^. The stretching vibration of the hydroxyl group on chitosan was seen at 1096 cm^−1^, with the stretching vibration of the iron oxide bidentate ligand occurring at 891 cm^−1^, and 794 cm^−1^ represented the in-plane and out-of-plane bending vibration of Fe-OH on magnetite. This indicated that the composite combined the active functional groups of magnetite and chitosan.

Therefore, the structure of magnetite in the composite remained unchanged, while the active functional groups of chitosan were still present. The compounding process did not alter the chemical properties of the two components, thus necessitating the consideration of the contributions of both components when examining the adsorption mechanism.

**Figure 3:**
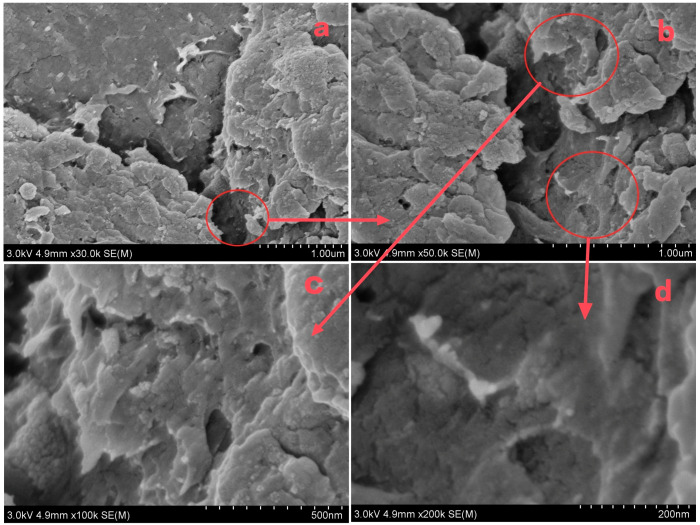
SEM images of magnetite–chitosan composite ((**a**–**d**) represent the 30,000-, 50,000-, 100,000- and 200,000-times magnification of the view, respectively, and the red circle and arrow indicate the magnified field of view).

**Figure 4:**
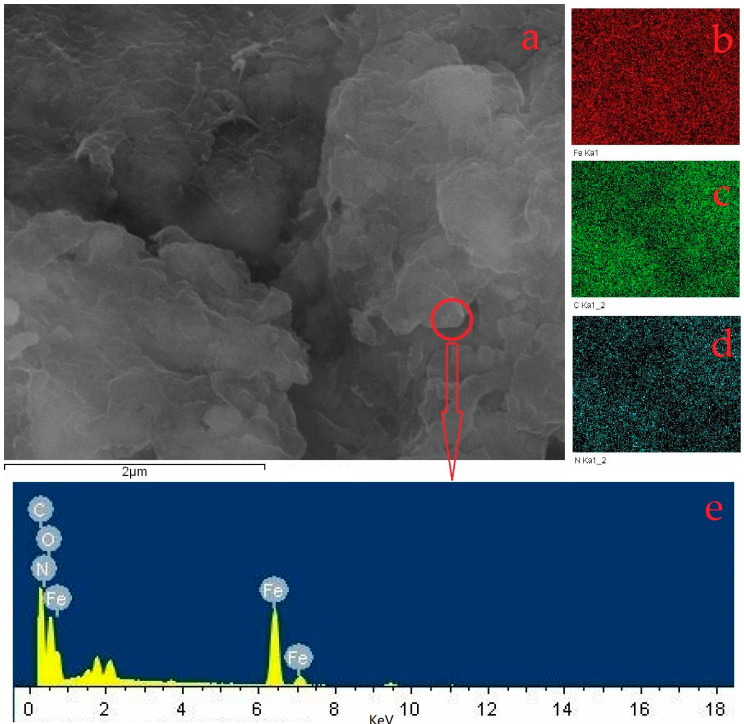
EDS analysis of the composite ((**a**), the field of the energy spectrum analysis; (**b**–**d**) are the elemental analysis of Fe, C and N by field scan; (**e**) the elemental analysis by point scan in the red circle in (**a**)).

From the morphology of the composite in Figure 3, it can be seen that the composite had a relatively rough surface and no obvious porous structure. No magnetite crystallization was observed at that magnification. The brighter region in Figure 3d showed chitosan (non-conductive). Hence, the other dark regions in the figure were likely to be composite structures of chitosan and magnetite. Figure 4a depicted the element surface scan which displayed the most uniform distribution of Fe (Figure 4b). Additionally, C and N (Figure 4c,d) exhibited distributions that correlated to the thickness shown in Figure 4a. An elemental composition analysis of the red circle area in Figure 4e revealed the presence of Fe, C, N and O with respective proportions of 40.02%, 23.22%, 33.11% and 3.66%. On the whole, the composite of magnetite and chitosan appeared to be relatively homogeneous.

### 3.2. The Effect of pH on the Adsorption

**Figure 5:**
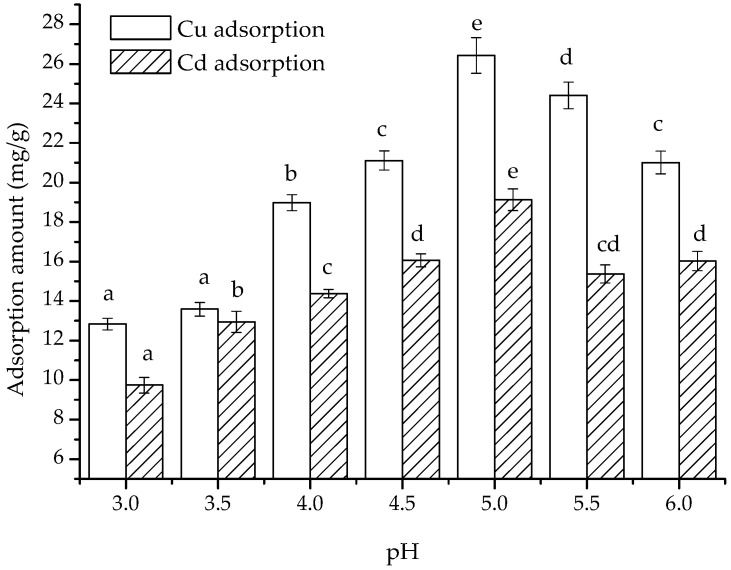
pH dependence of the adsorption of Cu(II) and Cd(II) on the composite. (The error bars showed the absolute errors in repeats of adsorbed amount; the letters a–e in the figure were the attribution subsets corresponding to the significance analysis results, Duncan, *p* < 0.01).

Significant changes in the adsorption of Cu(II) and Cd(II) on the composite were observed with changes in pH (Figure 5). At pH 3, the adsorbed amounts of Cu(II) and Cd(II) on the composite were 12.83 and 9.74 mg/g, respectively. As the pH increased from 3.5 to 5.0, the adsorbed amounts of Cu(II) and Cd(II) increased from 13.59 and 12.95 mg/g to 26.43 and 19.13 mg/g, respectively. However, when the pH was at 5.5 and 6.0, the adsorbed amounts of Cu(II) decreased to 24.41 and 21.01 mg/g, whereas those of Cd(II) decreased to 15.37 and 16.03 mg/g, respectively. This result is consistent with the previous observation that the highest adsorption occurred at pH 5 [30] but not with the conclusion that the maximum adsorption capacity occurred at pH 6.0 [15], which may be attributed to the differences in components.

Chitosan has the *pK_a_* at 6.5 [34], so it was pronated when the pH was below 6.5, which was enhanced by the acid, resulting in increased adsorption of cations. On the other hand, a lower pH provided more H^+^, which competed for the binding sites of the cations. Based on changes in adsorption amount, we postulate that when the pH is less than 5, competitive adsorption enhancement by the hydrogen ions outweighs the enhancement in adsorption capacity due to protonation of chitosan. In contrast, at pH values above 5, weakening of chitosan protonation outweighs the decreasing competitive adsorption of hydrogen ions, resulting in the maximum adsorption amount at pH 5 in the experimental pH range, while adsorption amount diminished under any other pH conditions. pH values greater than 6 may lead to cation precipitation and were thus not considered.

### 3.3. Isothermal Adsorption and Fitting Analysis

**Figure 6:**
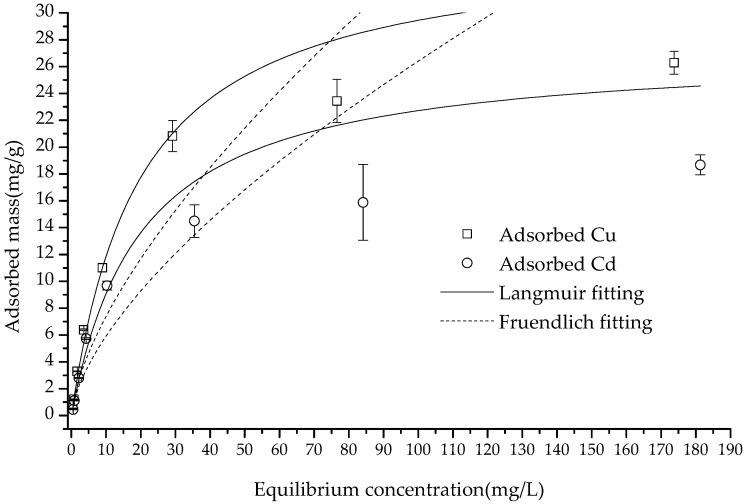
The isotherm adsorption of Cu(II) and Cd(II) on the composite and the fitting with the equations.

**Figure 7:**
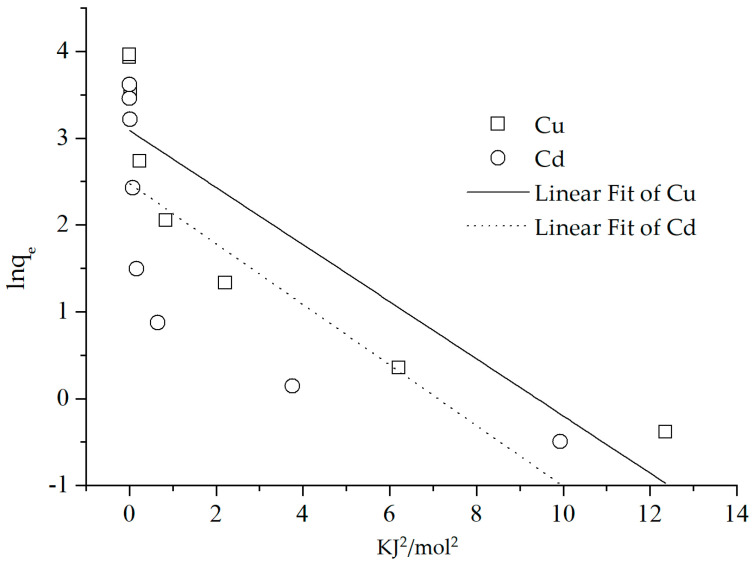
The fitting with D–R equation.

As shown in Figure 6, within the equilibrium concentration range of 0–200 (mg/L), the adsorption amount of Cu(II) and Cd(II) increased in correlation with the increase in the concentration. When the equilibrium concentrations were 174.41 and 181.52 mg/kg, respectively, the adsorbed Cu(II) and Cd(II) values reached their maxima of 26.28 and 18.67 mg/g, almost reaching a saturation point. According to the calculation with formulas (1)–(6), the correlation coefficients of the Langmuir equation, which was *R*^2^ = 0.9266 and 0.9323 for the adsorption of Cu(II) and Cd(II), were much higher than those of the Freundlich equation and D–R equation—*R*^2^ = 0.6524, 0.6556, 0.7408 and 0.5401, respectively (Figure 7 and Table 1). The higher fitting of the Langmuir equation showed that the adsorption of these two cations was monolayer adsorption, and the process was controlled by a single category of adsorption [35]. Furthermore, the Langmuir equation fitting demonstrated that the composite had a theoretical maximum adsorption capacity (*q_m_*) of 35.26 and 27.27 mg/g for Cu(II) and Cd(II), respectively, which was much greater than the observed data.

The measured maximum adsorbed Cu(II) and Cd(II) were lower than those reported for nano magnetite–chitosan films (123.4 mg/g Cu and 112.3 mg/g Cd) [26], chitosan–calcium alginate–bentonite (115.30 mg/g Cu and 102.38 mg/g Cd) [36], and kaolinite-immobilized chitosan beads (88.5 mg/g Cd) [15]. Nevertheless, the Cu(II) adsorption was greater than the reported 21.034 mg/g on expectedly monodisperse chitosan-bound Fe_3_O_4_ magnetic nanoparticles [37]. This may be accounted for due to differences in the effective surface of various adsorbents. The dimensionless factor *R_L_* was found to be lesser than 1 at each initial concentration, thereby indicating that the composite’s adsorption of Cu(II) and Cd(II) was favorable under the experimental conditions. The intensity *1*/*n* of the two cations on the adsorbent was less than 1, indicating that the adsorption of Cu(II) and Cd(II) by the composite tended to be irreversible under the experimental conditions [5,38]. The poor fitting of the Freundlich equation precluded the meaningful discussion of the fitting parameter *K_F_*. On the other hand, the *E* value obtained from the D–R equation suggested that the adsorption was likely dominated by physisorption [39,40]. However, the low correlation values (0.7408 and 0.5401) coupled with the non-porous structure of the composite, as indicated by the aforementioned SEM, pointed to a combination of both physisorption and chemisorption as the likely process.

Since chitosan has an 80% degree of deacetylation, 1 kg of chitosan contains 4.112 moles of amino groups (the molecular weights of deacetylated and non-deacetylated monomers are 161 and 203 g/mol, respectively). The number of binding sites with free amino groups on the chitosan was far more than the charge of magnetite (at pH 7), so it can be inferred that the binding sites of the composite were mainly attributed to the amino groups of chitosan [38].

Generally, the greater the differences in electronegativity between N (or O) and metal ions are, the stronger the adsorption attraction is. The electronegativity of Cu(II) and Cd(II) was 2.98 and 2.71, respectively, which could explain why the adsorption amount of Cu was higher than that of Cd [9].

### 3.4. Kinetic Adsorption and Fitting Analysis

**Figure 8:**
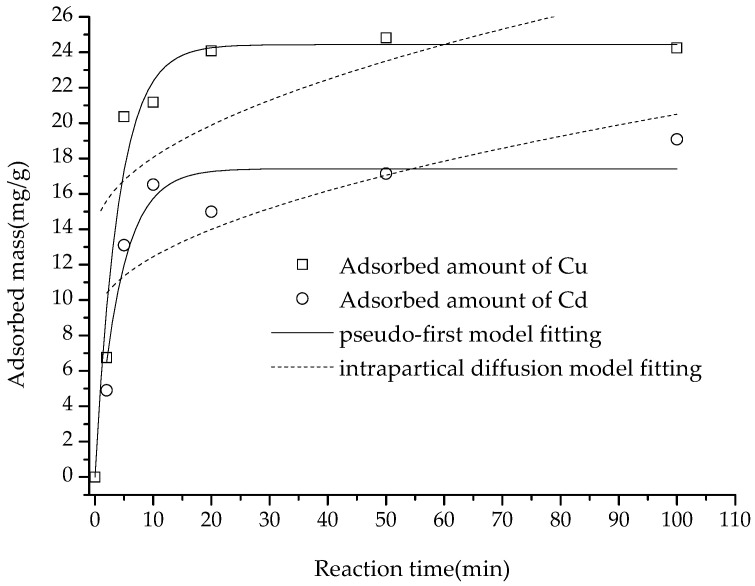
Adsorption of Cu(II) and Cd(II) on the composite versus time increasing and the fitting with pseudo-first and intraparticle diffusion equations.

As shown in Figure 8, the adsorption amount of Cu(II) and Cd(II) increased with the increase in interaction time. Cu(II) and Cd(II) adsorption capacities were 21.18 and 16.52 mg/g at 10 min reaction time, respectively, and reached the maximum adsorption capacity of 24.25 and 19.08 mg/g at 90 min. The time taken to reach equilibrium in this study was far shorter than the 60 min [5] and 120 min [26] reported in the literature, which might be attributed to the fact that the adsorbent in this study had fewer inner surfaces, which might also explain the smaller amount of adsorption.

**Figure 9:**
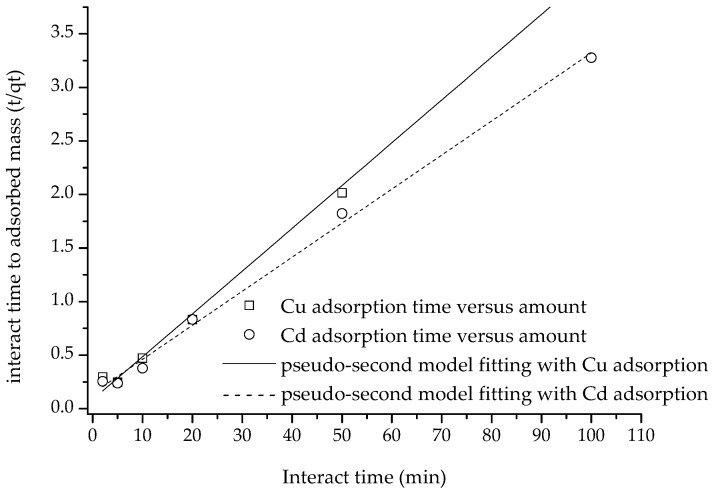
The fitting of kinetic adsorption with pseudo-second equation.

After fitting the adsorption amount data to the pseudo-first-order, pseudo-second-order and intra diffusion models, it was discovered that the pseudo-second-order equation was the best fit for the adsorption data of the two heavy metal ions (with *R*^2^ values of 0.9969 and 0.9955, respectively), followed by the pseudo-first-order equation (*R*^2^ values of 0.9620 and 0.8777, respectively), and the intra-particle diffusion equation was least successful (with *R*^2^ values of 0.2971 and 0.4766, respectively) (Figure 8 and Figure 9).

According to the literature, there is a consensus that pseudo-first-order, pseudo-first-order and intra-particle diffusion equation (pseudo-third-order) have different meanings. It is well-established that a higher *R*^2^ value implies that physical adsorption, chemisorption and internal surface diffusion are the rate-limiting steps for these three equations, respectively [29,30,41]. Thus, adsorption of Cu(II) and Cd(II) was determined to be of a chemisorptive nature, suggesting that chemisorption was the rate-limiting factor of the adsorption process. In contrast, the results showed that internal diffusion was not the rate-limiting stage of adsorption [29], indicating that the composite did not have a significant inner surface, leading to easy access for cations to its reactive sites.

### 3.5. Thermodynamic Adsorption and Parameter Calculation

**Figure 10:**
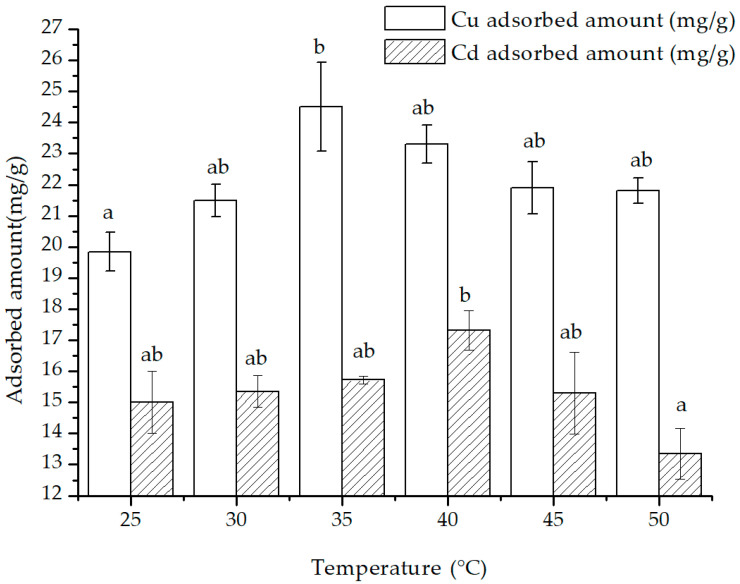
The effect of temperature on the adsorption of Cu(II) and Cd(II) (the letters a and b in the figure were the attribution subsets corresponding to the significance analysis results, Duncan, *p* < 0.05).

With the rise in temperature, the adsorbed amount of Cu(II) and Cd(II) showed an increasing trend that later declined (Figure 10). From 25 °C to 35 °C, the adsorption of Cu(II) and Cd(II) increased from 19.85 to 24.51 mg/g and from 15.01 to 15.72 mg/g, accordingly. However, as the temperature went beyond 35 °C and up to 50 °C, the adsorption of both Cu(II) and Cd(II) decreased to 21.81 and 13.35 mg/g, respectively. Subsequently, the enthalpy, entropy and free energy change values of the thermodynamic adsorption were computed and presented in Table 2 in accordance with formulas (10)–(12).

The Δ*G*_0_ of both Cu(II) and Cd(II) adsorption was positive in the temperature range of 25 °C to 50 °C, implying that the adsorption of the two cations onto the composite cannot occur spontaneously [42]. Lower Δ*G*_0_ values indicated that the driving force for adsorption was stronger [31]. The Δ*G*_0_ for Cd^2+^ adsorption was greater than that of Cu(II), indicating that Cu(II) was easier to be adsorbed than Cd(II) within the experimental temperature range. Hence, Cu(II) had more adsorption benefits than Cd(II) on the surface of the composite.

A positive value of Δ*H*_0_ implies that the adsorption process is endothermic, while a negative Δ*H*_0_ would suggest an exothermic reaction. Similarly, a positive value of Δ*S*_0_ signifies that there is an increase in the disorder of heavy metal ions on the surface, indicating that the adsorption process is entropy-driven. It was calculated that the Δ*H*_0_ of adsorption of Cu(II) and Cd(II) within the range of 25 °C to 50°C was −3.9215 and −2.9497 KJ/mol, respectively, while their Δ*S*_0_ was 1.2896 and −0.0163 J·mol/K.

Considering that the adsorption amount increased at first and then decreased with the increasing temperature, the significance of the enthalpy and entropy change in the whole process was limited, and the enthalpy and entropy changes were calculated for the two temperature ranges of 25 °C to 35 °C and 40 °C to 50 °C, respectively (Table 2). In the temperature range of 25 °C to 35 °C, the Δ*H*_0_ and Δ*S*_0_ for the adsorption of both Cu(II) and Cd(II) were positive, indicating that the adsorption reactions were endothermic, the disorder of adsorption increased, and the adsorption was an entropy-driven process. Conversely, for the range of 40 °C to 50 °C, the Δ*H*_0_ and Δ*S*_0_ values of Cu(II) and Cd(II) adsorption were both negative, indicating the adsorption mechanism for these two cations might change. These reactions became exothermic, the randomness of the adsorption was reduced, and a fixed adsorption mechanism dominated the adsorption process..

The compositional properties of the adsorbent composed of magnetite and chitosan did not significantly change across the temperature range in the present study. Based on their size, their composite should be similar to the relationship between ropes and pendants. Research and the literature [6,31,37] indicate that the chitosan chain might expand with an increase in temperature. This change in state causes the chitosan and/or magnetite exposed more functional groups to take part in the adsorption of heavy metal cations. This could explain the two-stage variation of the adsorption amount and calculation parameters observed, implying that bonding forms of magnetite and chitosan are distinct, different temperature conditions, thus affecting the adsorption mechanism.

### 3.6. Regenerative Cycle Adsorption

**Figure 11:**
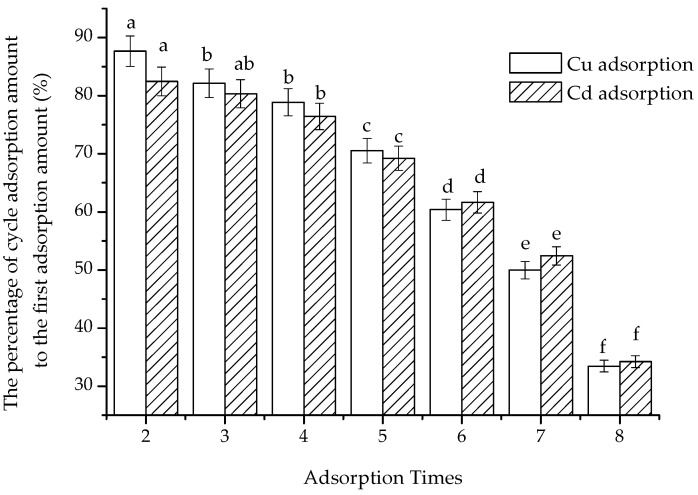
The adsorption amount after the regeneration (the letters a–f in the figure were the attribution subsets corresponding to the significance analysis results, Duncan, *p* < 0.01).

As can be seen from Figure 11, the adsorption amount of the regenerated composite significantly decreased with regeneration times. After the first regeneration, the adsorption capacity of Cu(II) and Cd(II) dropped to 87.67% and 82.45% of the initial adsorption, respectively. Following the second regeneration, the third adsorption capacity of Cu(II) and Cd(II) was reduced to 82.15% and 80.34%, respectively. After the third regeneration, the fourth adsorption capacity fell below 80% of the initial adsorption. Furthermore, the adsorption amounts of Cu(II) in the sixth, seventh and eighth regenerations were 60.37%, 49.98% and 33.45%, respectively, and those of Cd(II) were 61.65%, 52.43% and 34.21%, respectively, compared to the first adsorption.

The adsorption capacity of the composite remained at over 80% even after two regenerations and at 60% after five regenerations. The regeneration efficiency was lower than the reported 95% [26]. This discrepancy could be attributed to the agents that were added and the variance in the structure of the composite [15]. Spherical chitosan nanoparticles encapsulated the cubic-shaped magnetic iron oxide particles; different components and ratios of the composite could lead to different levels of stabilities [24]. In this study, the capacity attenuation was relatively noticeable. This might have been a result of the loss of chitosan that took place during the regeneration process.

### 3.7. Comparison of the Other Adsorption

As shown in Table 3 (Reference [4] provided that the removal rate of 100 mg/L Cu(II) by 100 mg/L adsorbent was 61.44–93.39%, which was used to calculate the adsorption capacity), a few studies had reported on the adsorption properties of the magnetite–chitosan-based composite, including modification such as addition of kaolinite, ferric chloride and modifiers or crosslinking agents, such as acryl-amide and glutaraldehyde. There have also been some exploratory modifications to the morphology of the composite, such as microspheres and membranes. Judging from the literature presented in the table, the adsorption amount of Cu(II) in this study was only slightly higher than 21.5 mg/g [37], which was lower than all other studies. In comparison, the time to reach adsorption equilibrium in this study was only longer than the conclusion of one literature [37] and shorter than all other literature.

The results of this study showed that the recycling performance was worse than what had been reported in the other literature. It could only maintain a regeneration rate of 80% after two regenerations, compared to the highest regeneration rate of 95% reported after five regenerations, indicating that the stability of the composite at present was limited, and glutaraldehyde proved to be an effective crosslink agent.

On the other hand, without incorporating any cross-linking agent, this study demonstrated relatively general adsorption capacity and regeneration stability (the capacity of around 20 mg/g and an 80% regeneration rate still possessed considerable application potential). Notably, the observed faster adsorption rate compared to other studies without a cross-linker was likely due to the composite’s more straightforward structure with fewer inner surfaces.

## 4. Conclusions

In this study, (1) no grafting or modified materials were used in the synthesis of the magnetite–chitosan composite; nonetheless, it showed a good performance in adsorbing heavy metals (Cu(II) and Cd(II) at 26.28 and 18.67 mg/g, respectively); (2) while its adsorption capacity was lower than some previously reported results, its equilibrium time (10 min) was shorter than most of the literature, potentially due to the composite’s simple structure; (3) the adsorption of cations adsorption increases from 25 °C to 35 °C and decreased from 40 °C to 50 °C. The chitosan on the composite material may expand with the increases, thereby altering the adsorption mechanism of cations; (4) the composite has a comparatively rough exterior, but its inner surface and porosity are not conspicuous due to the combination of magnetite and chitosan present, with chitosan likely dominating the adsorption.

This implies that the potential of natural composites remains abundant. Where grafting and modification cannot be used, exploring this environment-friendly and pure material should be ongoing. Additionally, further studies should be conducted to investigate the structural changes and various adsorption properties of the magnetite–chitosan composite under different temperature conditions. This organic–inorganic natural composite has great potential for pollutant passivation-adsorption without modification, which is quite promising.

## Figures and Tables

**Table 1:** The parameters of the fitting with Cu(II) and Cd(II) adsorption.

	Langmuir					Freundlich			D–R		
	*R* ^2^	*q_m_* (mg/g)	*b*	*C*_0_ (mg/L)	*R_L_*	*R* ^2^	*K_F_* (mg/g)	1/*n*	*R* ^2^	*q_e_* (mg/g)	*E* (KJ/mol)
Cu	0.9266	35.2648	0.0506	1–200	0.09–0.95	0.6524	1.6041	0.6624	0.7408	21.9463	1.2333
Cd	0.9323	27.2670	0.0499	1–200	0.09–0.95	0.6556	1.3210	0.6500	0.5401	11.9377	1.1976

**Table 2:** The thermodynamic parameters of Cu(II) and Cd(II) on the composite.

Temperature (°C)	Δ*G*^0^ (KJ/mol)		Δ*H*^0^ (KJ/mol)		Δ*S*^0^ (J·mol/K)	
	Cu(II)	Cd(II)	Cu(II)	Cd(II)	Cu(II)	Cd(II)
25	1.0365	2.0958	28.8740	5.0566	0.0932	0.0099
30	0.7128	2.0516				
35	0.1008	1.9964				
40	0.3549	1.6519	−10.1450	−31.4900	−0.0337	−0.1059
45	0.6606	2.1659				
50	0.6888	2.7109				

**Table 3:** Comparison of other adsorbents reported in the literature for the removal of Cu(II) and Cd(II).

Different Component	Modification/Cross-Linking	Form	Metal	Capacity (mg/g)	Equilibrium Time	Recovery Rate	References
Carboxymethyl chitosan	acryl-amide, 2-acrylamide-2-methylpropanesulfonic acid	nanoparticle	Cu(II)	614.4–933.9	80 min	77% at 5 times	[4]
None	glutaraldehyde	film	Cu(II) Cd(II)	123.4, 112.3	120 min	95% at 5 times	[26]
Carboxymethyl chitosan	carbodiimide	nanoparticles	Cu(II)	21.5	1 min		[37]
kaolinite		bead	Cd(II)	88.5	60 min	80% at 4 times	[15]
None		nanoparticle	Cu(II); Cd(II)	26.28, 18.67	10 min	80% at 2 times	present

## Data Availability

Data are contained within the article.
